# High Body Mass Index Is Associated with an Increased Risk of the Onset and Severity of Ossification of Spinal Ligaments

**DOI:** 10.3389/fsurg.2022.941672

**Published:** 2022-07-22

**Authors:** Yongzhao Zhao, Qian Xiang, Jialiang Lin, Shuai Jiang, Weishi Li

**Affiliations:** ^1^ Department of Orthopaedics, Peking University Third Hospital, Beijing, China; ^2^ Beijing Key Laboratory of Spinal Disease Research, Beijing, China; ^3^ Engineering Research Center of Bone and Joint Precision Medicine, Ministry of Education, Beijing, China

**Keywords:** ossification of the ligamentum flavum, ossification of the posterior longitudinal ligament, body mass index, onset, severity, systematic review, meta-analysis

## Abstract

**Background:**

Ossification of the posterior longitudinal ligament (OPLL) and that of ligamentum flavum (OLF) are the main types of the ossification of spinal ligaments (OSL) that cause the thoracic myelopathy. Although several studies have investigated the relationship of body mass index (BMI) with the onset or severity of OSL, it remains unverified due to the contradictory results of existing evidence. A systematic review and meta-analysis were performed in this work to determine the relationship of BMI with the onset and severity of OSL.

**Methods:**

PubMed, EMBASE, Web of Science, and Cochrane Library were comprehensively searched online for relevant studies focusing on the relationship of BMI with the onset or severity of the OSL. The difference in BMI of OSL (or severe OSL group) and non-OSL (or nonsevere OSL group) groups was evaluated using the mean difference (MD) with a corresponding 95% confidence interval (CI).

**Results:**

Fifteen studies were included in this systematic review and meta-analysis. The BMI of the OSL group was significantly higher than that of the non-OSL group (MD = 1.70 kg/m^2^, 95% CI = 1.02–2.39 kg/m^2^, and *P* < 0.01). Similar results were observed in the subgroup analysis of female (*P* < 0.01), OPLL (*P* < 0.01), and OLF (*P* < 0.01) populations. Three studies reported a significant association of BMI with the ossification index of OSL and the standardized regression coefficient ranging from 0.11 to 0.43 (*P* < 0.05). Moreover, a significantly higher BMI was observed in the severe OSL group compared with that in the nonsevere OSL group (MD = 3.09, 95% CI, 0.22–5.97 kg/m^2^, and *P* = 0.04).

**Conclusion:**

The significant association of high BMI with the onset and severity of OSL may provide new evidence and insights into the mechanism research and management of OSL.

## Introduction

Ossification of spinal ligaments (OSL) is a characterized heterotopic disease that can lead to severe myelopathy by compressing the spinal cord (1). Ossification of the posterior longitudinal ligament (OPLL) and ossification of the ligamentum flavum (OLF) are common and clinically significant types of OSL that generally progress and easily result in spinal injury; meanwhile, patients diagnosed with OPLL or OLF often complain of the local neck or thoracic pain and stiffness, difficulty in walking, limb weakness, and even incontinence (2, 3).

The precise pathogenesis of OSL remains unclear, and OSL is widely thought to be due to a mix of genetic and environmental factors (2–4). OSL typically occurs in Asians, especially the East Asian population, thereby indicating that genetic factors may play a vital role in its onset; however, many environmental factors, such as mechanical stress, metabolic disorders, gender, and age, may also contribute to the occurrence of OSL (5, 6). A few researchers have recently begun to focus on the relationship of obesity or body mass index (BMI) with the onset or severity of OSL because the clinical practice has presented the susceptibility of OSL in the obese population, although the results of existing studies are contradictory (6–20). Kobashi et al. (6) showed that the BMI of the OPLL group is significantly higher than that of the non-OPLL group in both male (25.8 kg/m^2^ vs. 23.3 kg/m^2^, *P* < 0.05) and female (25.9 kg/m^2^ vs. 23.4 kg/m^2^, *P* < 0.05) individuals. Similarly, Mori et al. (15) observed a significantly higher BMI in the OPLL group compared with that in the non-OPLL group (24 kg/m^2^ vs. 22 kg/m^2^, *P* < 0.05). However, Shirakura et al. (18) demonstrated that the BMI difference between OSL and non-OSL groups is nonsignificant (*P* > 0.05). Meanwhile, Feng et al. (12) also failed to observe the distinct difference in the BMI between OPLL and non-OPLL groups in both male (*P* > 0.05) and female (*P* > 0.05) individuals. Hirai et al. (10) observed a positive relationship between the BMI and ossification index in OPLL patients (*P* < 0.05) with consideration for the severity of OSL, thereby indicating that high BMI may increase the severity of OPLL. Similarly, Endo et al. (9) demonstrated there was a significantly higher BMI in patients with multilevel OLF compared with that in patients with localized OLF (*P* < 0.05). By contrast, Ikeda et al. (11) showed there was no significant difference in BMI between OPLL limited at the cervical spine and cervical OPLL extended to the thoracic or lumbar spine (*P* > 0.05). Therefore, the role of BMI in the onset and severity of OSL remains unverified. BMI is a typical measurement for the assessment of obesity, which is a very common metabolic disease that can be successfully managed with diet control and exercise (21). Obesity is associated with several human diseases, such as tumors, diabetes, and osteoarthritis (22–24). However, studies on the definite character of obesity in OSL are lacking. Hence, determining the role of BMI in the pathogenesis of OSL is essential in the prevention and treatment of this disease.

We performed a systematic review and meta-analysis in this study to determine the relationship of BMI with the onset and severity of OSL by integrating existing evidence. We speculated that high BMI could increase the risk of onset and severity of OSL.

## Materials and Methods

This systematic review and meta-analysis was strictly conducted according to Preferred Reporting Items for Systematic Reviews and Meta-Analyses (25) and registered in PROSPERO (https://www.crd.york.ac.uk/prospero/) (CRD42021289323). This study has been approved by the Ethics Committee of Peking University Third Hospital. The written informed consent was not necessary because all data were extracted from published studies.

### Eligibility Criteria for Study Selection

Original investigations were eligible for inclusion if they reported data on the association of BMI with the onset or severity of OSL regardless of their study design. The following studies were directly excluded from this research: cadaver experiments, case reports, reviews, animal or cell experiments, non-English reports, and investigations containing duplicate patients for statistical analysis.

### Information Source, Literature Search, and Study Selection

PubMed, EMBASE, Web of Science, and Cochrane Library were searched online on June 1, 2022 using the following keywords: “ossification of yellow ligament,” “ossification of ligamentum flavum,” “ossification of posterior longitudinal ligament,” “OPLL,” “ossification of the spinal ligament,” “BMI,” “body mass index,” and “obesity.” Details on the literature search in the PubMed database are listed in Supplementary Table S1. Eligible studies were then independently selected by two authors according to the eligibility criteria, and any disagreement was resolved by group discussion.

### Data Collection

The following items were extracted from all included studies that met the inclusion criteria: authors' name, published year, country, research center, sample size, gender and age of patients, types of OSL, location of OSL, recruitment time of patients, and relevant data regarding the relationship of BMI with the onset or severity of OSL.

### Risk of Bias in Individual Studies

The Newcastle–Ottawa scale (NOS), an assessment tool for cohort and case–control studies, was utilized in this work to evaluate the risk of bias in included studies (26). The NOS contains three main categories with a maximum of nine points, including the selection, comparability, and ascertainment of outcomes. NOS scores with 0–3, 4–6, and 7–9 stars were defined as high risk, moderate risk, and low risk, respectively (26).

### Statistical Analysis

All analyses were performed using Review Manager 5.3 (Cochrane Collaboration, London, UK) and Stata 12.0 (StataCorp, College Station, TX). The difference in BMI between OSL (severe OSL group) and non-OSL (nonsevere OSL group) groups was evaluated using the mean difference (MD) with a confidence interval (CI) of 95%. Heterogeneity across the included studies was assessed using the chi-squared test, and a random-effects model was applied for the evident heterogeneity (*P* < 0.10, I^2^ ≥ 50%). Otherwise, a fixed-effect model should be used. Subgroup analyses stratified by gender and types of OSL were also performed to determine the association between BMI and onset of OSL further. Sensitivity analysis was conducted to check the stability of the pooled results. Publication bias among the included studies was assessed using Begg's and Egger's tests. A *P* value less than 0.05 indicated that the difference is statistically significant.

## Results

### Characteristics of the Included Studies

**Figure 1:**
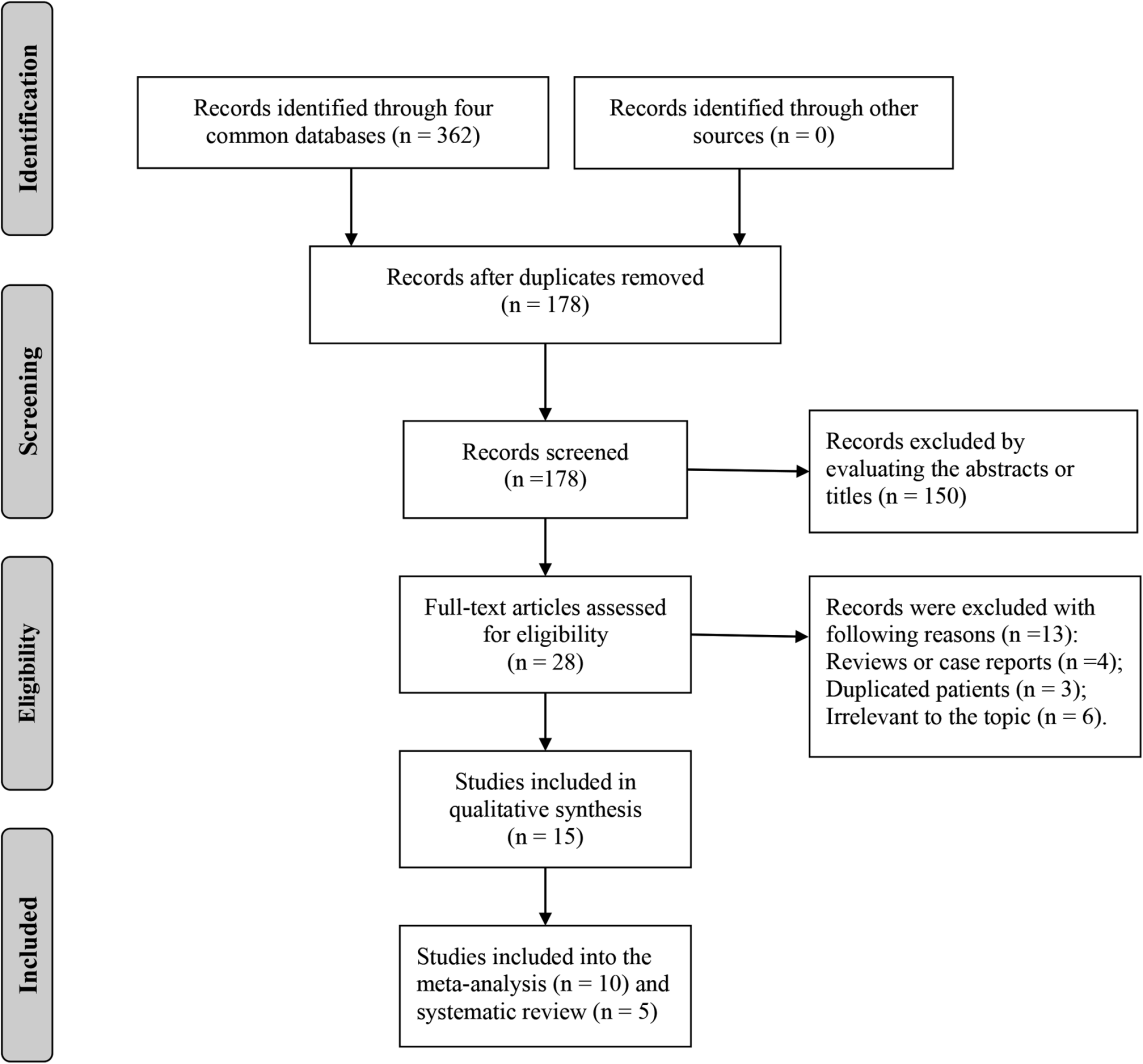
Flow diagram of literature search and selection.

A total of 362 records were retrieved from PubMed, EMBASE, Web of Science, and Cochrane Library, and 15 studies were finally included in the systematic review and meta-analysis of this study (Figure 1) (6–20). Characteristics of the included studies are listed in Table 1. Ten studies focused on the OPLL (6, 7, 9–12, 14–17) and four studies focused on the OLF (9, 13, 19, 20) with respect to the types of OSL. Twelve studies containing 24,710 individuals explored the association between the BMI and onset of OSL (6, 9, 11–20), and five studies on 797 OSL patients explored the association between the BMI and severity of OSL (7–11). Notably, Ikeda et al. (11) and Endo et al. (9) both simultaneously analyzed the relationship of BMI with the onset and severity of OSL. Five studies with a NOS score of 7 were considered low risk (8, 10, 12, 13, 20) and ten studies with a NOS score of 6 were considered moderate risk (6, 7, 9, 11, 14–19) in terms of the risk of bias. The NOS details for each study are listed in Supplementary Table S2.

**Table 1:** Characteristics of included studies about the relationship of BMI with the onset of OSL.

Study	Country	Research center	Sample size (total/OSL) (*n*)	Gender (male/female) (*n*)	Age (OSL/non-OSL)(mean, year)
Shingyouchi 1996 (17)	Japan	Single center	4,802/198	NA	51.41
Shirakura 2000 (18)	Japan	Single center	106/49	69/37	59.26/59.95
Kobashi 2004 (6)	Japan	Multiple center	207/69	120/87	61.71/61.77
Ikeda 2011 (11)	Japan	Single center	187/125	103/84	60.01/58.76
Mori 2014 (15)	Japan	Single center	3,013/56	1,752/1,261	68/65
Feng 2018 (12)	China	Single center	64/35	39/25	58.48/59.60
Kim 2018 (13)	South Korea	Single center	4,999/1,090	2,929/2,070	60.9/60.9
Chang 2020 (20)	South Korea	Single center	129/43	69/60	69.5/69.0
Liao 2020 (14)	China	Single center	7,210/1,314	4,546/2,664	54.00
Oshima 2020 (16)	Japan	Single center	1,789/120	1,116/673	63.3 /58.2
Endo 2021 (9)	Japan	Multiple center	340/204	190/150	52.6/64.95
Tang 2021 (19)	China	Multiple center	1,864/114	1,158/706	47.5
Study	BMI (OSL/non-OSL)	Types of OSL	Location of OSL	Recruitment time	NOS
Shingyouchi 1996 (17)	Incidence of OPLL: BMI ≥ 25 kg/m^2^ group (6.0%), BMI < 25 kg/m^2^ group (3.2%), *P* < 0.01	OPLL	Cervical spine	1984–1994	6
Shirakura 2000 (18)	BMI: male: 23.3 ± 0.6/23.7 ± 0.5 kg/m^2^, NS; female: 25.4 ± 1.1/23.4 ± 0.4 kg/m^2^, NS	OSL	Whole spine	1999–2011	6
Kobashi 2004 (6)	BMI: male: 25.8 ± 0.6/23.3 ± 0.3 kg/m^2^, *P* < 0.05; female: 25.9 ± 0.9/23.4 ± 0.4 kg/m^2^, *P* < 0.05	OPLL	Cervical / thoracic spines	1998–2001	6
Ikeda 2011 (11)	BMI: male: 24 ± 2.7/23.1 ± 2.5 kg/m^2^, NS; female: 25.2 ± 4.4/22.9 ± 3.1 kg/m^2^, *P* < 0.05	OPLL	Whole spine	1995–2008	6
Mori 2014 (15)	BMI: male: 24 ± 3/22 ± 3.4 kg/m^2^, *P* = 0.01; female: 23 ± 4.2/22 ± 3.6 kg/m^2^, *P* = 0.06; total: 24 ± 3.9 /22 ± 3.5 kg/m^2^, *P* < 0.01	OPLL	Thoracic spine	Wthin 2010	6
Feng 2018 (12)	male: 26.47 ± 3.46/25.26 ± 4.46 kg/m^2^, NS; female: 25.49 ± 3.05/24.10 ± 3.37 kg/m^2^, NS	OPLL	Cervical spine	NA	7
Kim 2018 (13)	BMI: male: 23.2 ± 3.6/23.3 ± 3.5 kg/m^2^, NS; female: 23.4 ± 3.7/23.1 ± 3.6 kg/m^2^, NS	OLF	Thoracic spine	2014–2015	7
Chang 2020 (20)	BMI:26.04 ± 3.76/24.16 ± 3.22 kg/m^2^, *P* < 0.01	OLF	Thoracic spine	2014–2019	7
Liao 2020 (14)	BMI: 25.11 ± 3.13/24.26 ± 3.04 kg/m^2^, *P* < 0.01	OPLL	Cervical spine	2012–2016	6
Oshima 2020 (16)	BMI: 24.8/23.6 kg/m^2^, *P* < 0.01	OPLL	Cervical spine	Wthin 2011	6
Endo 2021 (9)	BMI: OLF + OPLL group: 30.4 ± 6.3 kg/m^2^ (*P* < 0.01), multiple OLF group:28.6 ± 4.8 kg/m^2^* (*P* < 0.01), localized OLF group: 23.1 ± 2.8 kg/m^2^ (NS) / non-OSL group: 23.3 ± 3.5 kg/m^2^	OLF	Thoracic spine	2015–2020	6
Tang 2021 (19)	incidence of new OLF: underweight group (BMI < 18.5 kg/m^2^) (30.8%); normal weight group (18.5–23 kg/m^2^) (42.7%); overweight group (BMI: 23–25 kg/m^2^) (52.8%)*; obesity group (BMI ≥ 25 kg/m^2^) (55.8%)*	OLF	Thoracic spine	2003–2006	6

*OSL, ossification of spinal ligaments; BMI, body mass index; NOS, Newcastle–Ottawa scale; OPLL, ossification of the posterior longitudinal ligament; OLF, ossification of the ligamentum flavum; NS, not significant (P > 0.05); NA, not available.*

### Relationship of BMI with the Onset of OSL

**Figure 2:**
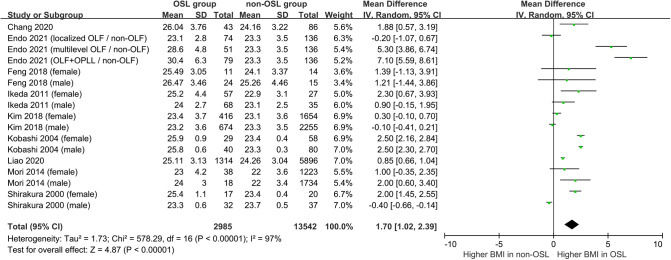
Meta-analysis of the relationship of BMI with the onset of OSL.

**Figure 3:**
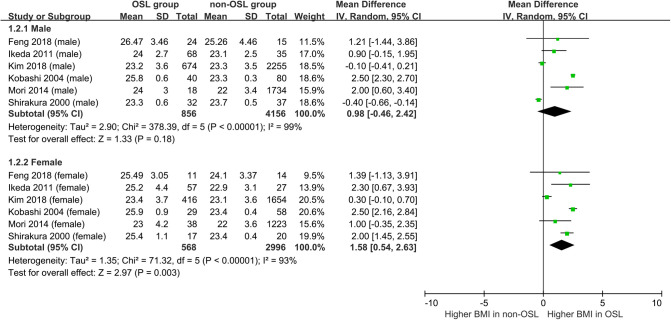
Subgroup analysis stratified by the gender for the relationship of BMI with the onset of OSL (*top*, males; *bottom*, females).

**Figure 4:**
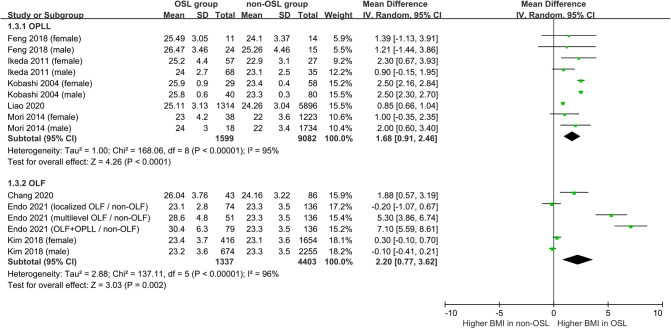
Subgroup analysis stratified by the type of OSL for the relationship of BMI with the onset of OSL (*top*, OPLL; *bottom*, OLF).

**Figure 5:**
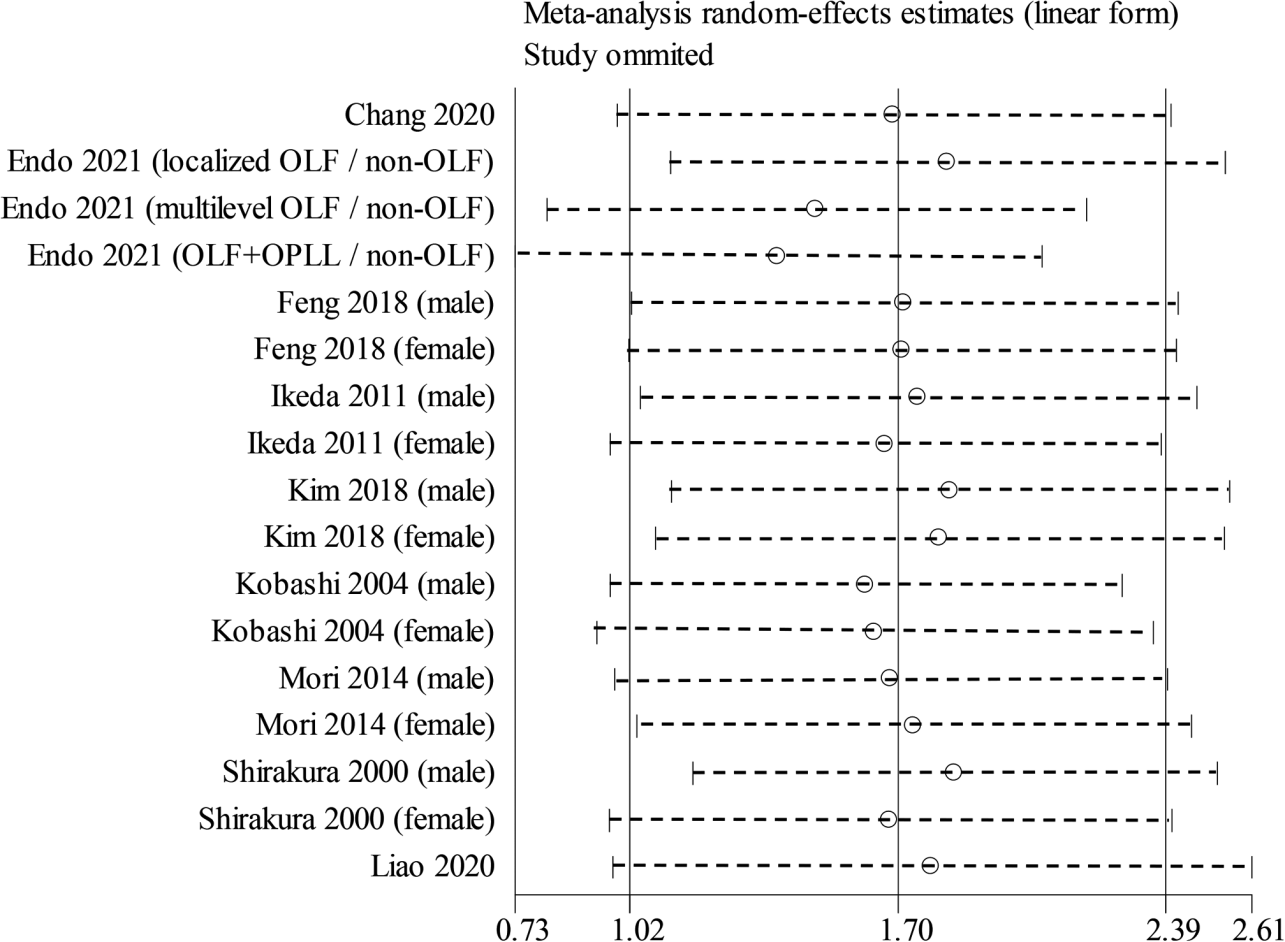
Sensitivity analysis for the relationship of BMI with the onset of OSL.

**Figure 6:**
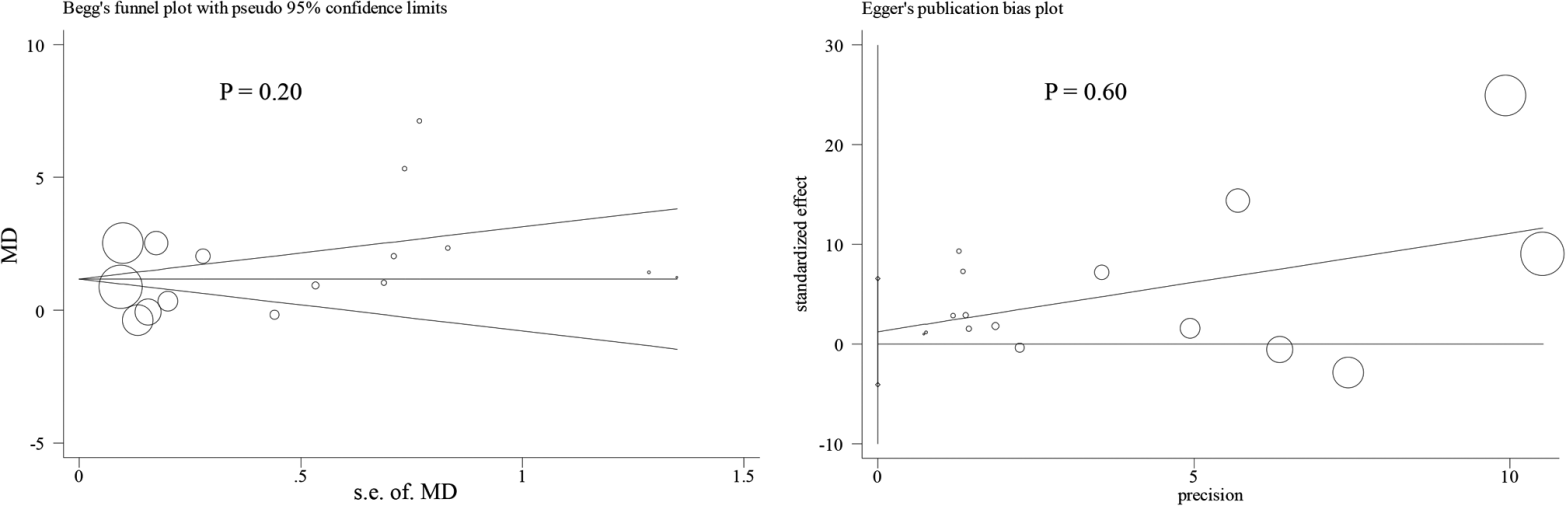
Publication bias across included studies (*left*, Begg's test; *right*, Egger's test).

Nine studies containing 17 case–control pairs that compared the BMI between OSL and non-OSL groups were included in the meta-analysis (6, 9, 11–15, 18, 20). The results showed that patients in the OSL group demonstrated a significantly higher BMI compared with those in the non-OSL group (*P* < 0.01), with an MD of 1.70 kg/m^2^ (95% CI and 1.02–2.39 kg/m^2^) (Figure 2). Subgroup analyses according to the gender and types of OSL were subsequently performed. The BMI of the OSL group was significantly higher than that of the non-OSL group in female individuals (MD = 1.58, 95% CI, 0.54–2.63 kg/m^2^, and *P* < 0.01) but nonsignificant in male individuals (MD = 0.98, 95% CI, −0.46–2.42 kg/m^2^, and *P* = 0.18) (Figure 3). The subgroup analysis based on types of OSL showed there was a significantly higher BMI is observed in both OPLL (MD = 1.68, 95% CI, 0.91–2.46 kg/m^2^, and *P* < 0.01) and OLF (MD = 2.20, 95% CI, 0.77–3.62 kg/m^2^, and *P* < 0.01) groups compared with that in the non-OSL group (Figure 4). Moreover, the results of the meta-analysis were considered stable according to the sensitivity analysis (Figure 5) and without evident publication bias among included studies based on Begg's (*P* = 0.20) and Egger's (*P* = 0.60) tests (Figure 6).

Three studies were excluded from this meta-analysis because of the different forms of data (16, 17, 19). Shingyouchi et al. (17) observed a significantly higher incidence of OPLL in the obesity group (BMI ≥ 25 kg/m^2^) compared with that in the nonobesity group (BMI < 25 kg/m^2^) (6.0% vs. 3.2%, *P* < 0.01). Tang et al. (19) also showed that new OLF typically occurs in obese (BMI ≥ 25 kg/m^2^) (55.8%) and overweight (23 ≤ BMI < 25 kg/m^2^) (52.8%) individuals compared with that in normal BMI (18.5 ≤ BMI < 23 kg/m^2^) (42.7%) and underweight (BMI < 18.5 kg/m^2^) (30.8%) (*P* = 0.02) individuals. Oshima et al. (16) reported that the BMI of OPLL individuals is significantly higher than that of non-OPLL individuals (24.8 kg/m^2^ vs. 23.6 kg/m^2^, *P* < 0.01).

### Relationship of BMI with the Severity of OSL

Five studies investigated the association between BMI and severity of OSL (7–11) (Table 2). Three studies assessed the relationship of BMI with the ossification index of OSL (7, 9, 10), which was calculated using the number of vertebral bodies and intervertebral disc level affected by the ossification site (27); meanwhile, the positive association of BMI with the ossification index of OPLL was observed using a standardized regression coefficient ranging from 0.11 to 0.43 in multiple regression analysis (*P* < 0.05) (7, 9, 10).

**Table 2:** Characteristics of included studies about the relationship of BMI with the severity of OSL.

Study	Country	Research center	Sample size (*n*)	Gender (male/female) (*n*)	Age (mean, year)
Akune 2001 (7)	Japan	Single center	100	89/11	58.10
Ikeda 2011 (11)	Japan	Single center	125	68/57	59.17
Hirai 2016 (10)	Japan	Multiple center	322	242/80	64.6
Ando 2017 (8)	Japan	Single center	46	25/21	56.01
Endo 2021 (9)	Japan	Multiple center	204	115/89	64.95
Study	Results	Types of OSL	Location of OSL	Recruitment time	NOS
Akune 2001 (7)	BMI was positively associated with the extent of ossification in multiple-regression analysis (*β* = 0.34, *P* < 0.01)	OPLL	Whole spine	1989–1999	6
Ikeda 2011 (11)	There was no significant difference in BMI between patients with OPLL limited to the cervical spine and those with OPLL extended to the thoracic and/or lumbar spine (male: 23.9 ± 2.6/24.3 ± 3.2 kg/m^2^, NS; female: 24.2 ± 5/25.5 ± 4.1 kg/m^2^, NS)	OPLL	Whole spine	1995–2008	7
Hirai 2016 (10)	(1) BMI is positively associated with the OP index in multiple regression analysis (*β* = 0.11, *P* < 0.05). (2) There was no significant difference in BMI between patients with cervical OPLL only and those with OPLL in multiple spinal regions (25.3 ± 4.4/26.1 ± 5.1 kg/m^2^, NS).	OPLL	Whole spine	NA	7
Ando 2017 (8)	There was a higher BMI in surgical therapy group compared with observational therapy (27.2/33.6 kg/m^2^, *P* = 0.02)	OPLL	Thoracic	2012–2015	7
Endo 2021 (9)	(1) Higher BMI in the OPLL+ OLF group (30.4 ± 6.3 kg/m^2^) and the multilevel OLF group (28.6 ± 4.8 kg/m^2^) compared with localized OLF (23.1 ± 2.8 kg/m^2^) (*P* < 0.01). (2) BMI was positively associated with the thoracic OLF index in multiple regression analysis (*β* = 0.43, *P* < 0.01)	OLF	Thoracic	2015–2020	6

*OSL, ossification of spinal ligaments; BMI, body mass index; NOS, Newcastle-Ottawa scale; OPLL, ossification of the posterior longitudinal ligament; OLF, ossification of the ligamentum flavum; NS, not significant (P > 0.05); NA, not available.*

**Figure 7:**
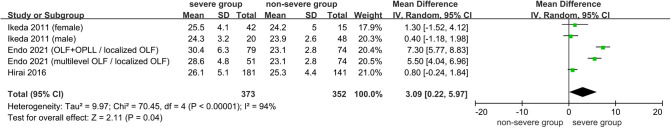
Meta-analysis for the relationship of BMI with the severity of OSL.

Three studies comparing the BMI between severe OSL and nonsevere OSL groups were included in the meta-analysis (9–11). The results showed that the BMI in the severe OSL group is significantly higher than that in the nonsevere OSL group (MD = 3.09, 95% CI, 0.22–5.97 kg/m^2^, and *P* = 0.04) (Figure 7). Ando et al. (8) also revealed that the BMI of patients treated with surgical therapy for severe myelopathy is significantly higher than that of patients treated with observation therapy for the absence of or mild myelopathy (33.6 kg/m^2^ vs. 27.2 kg/m^2^, *P* = 0.02); however, this study was excluded from the meta-analysis due to the lack of standard deviation of BMI.

## Discussion

OSL is a characterized heterotopic ossification disease with multiple potential risk factors likely associated with its pathogenesis (2, 3). Although several studies have explored the relationship between BMI and OSL, the results are conflicting and no definite conclusions have been obtained (6–20). Our study integrated the existing evidence and revealed that the BMI of the OSL group was higher than that of the non-OSL group (*P* < 0.01), thereby indicating that a high BMI may increase the risk of OSL. Moreover, a significant relationship existed between BMI and the ossification index of OPLL in three studies (*P* < 0.05), and the severe OSL group presents a higher BMI than the nonsevere OSL group (*P* = 0.04), thereby suggesting that a high BMI may aggravate the severity of OSL. Hence, our results showed that a high BMI would likely increase the onset and severity of OSL. To the best of our knowledge, this was the first study to perform a systematic review and meta-analysis to determine the relationship between BMI and the onset and severity of OSL.

We discovered that the significantly higher BMI of OSL individuals compared with that of non-OSL individuals might play an important role in the pathogenesis of this disease. Although previous studies indicated that a high BMI or obesity is involved in the pathology of several human diseases, such as tumors, diabetes, and cardiovascular diseases (28, 29), the underlying mechanism of a high BMI or obesity contributing to the pathogenesis of OSL remains unclear to date (12, 30). Leptin is secreted by adipose tissue and elevated in patients with OPLL and can promote the osteogenesis of OPLL cells *via* the ERK1/2, p38 MAPK, and/or JNK signaling pathways (12). Hyperleptinemia is a common characteristic of obese patients, and serum leptin is significantly increased in OPLL females compared to non-OPLL female controls (11). Similarly, hyperleptinemia was also observed in OLF patients, and the leptin-induced osteogenic effect in thoracic OLF cells may be associated with the activation of STAT3, JNK, and ERK1/2 (30). Furthermore, obesity is associated with a low-grade inflammation status, and adipose tissue can secrete several proinflammatory factors, such as C-reactive protein, tumor necrosis factor-α, and interleukin-6, which may exert crucial functions in the pathogenesis of OPLL or OLF (31, 32). It should be noted that although the OSL group typically exhibited a higher BMI than the non-OSL group in male individuals, this statistically nonsignificant difference requires further investigation. The following hypotheses are formed from these results: First, evident heterogeneity was observed across the included studies, and a random effect model may reduce the accuracy of the results. Second, the mean BMI of male individuals in the OSL group was low at 23.50 kg/m^2^, which may be insufficient to increase the risk of OSL. Additional studies should be conducted to determine the role of high BMI or obesity in the pathogenesis of OSL further.

We also explored the association of BMI with the severity of OSL, and three studies showed a positive association between BMI and the ossification index (*P* < 0.05), thereby indicating that high BMI likely promotes the severity of OSL (7, 9, 10). The results of our meta-analysis also presented that a high BMI is significantly associated with the severity of OSL. The association of high BMI with the severity of OSL may be interpreted by some published research studies (33, 34). Endo et al. (33) revealed the occurrence of a higher BMI in the early-onset OPLL group compared with that in the common OPLL group (34.2 kg/m^2^ vs. 25.6 kg/m^2^, *P* < 0.05). Katsumi et al. (34) reported that a high BMI is a potential risk factor for the annual rate of lesion increase in OPLL patients (*P* = 0.03); therefore, the higher severity of OSL in patients with higher BMI may be due to the earlier onset and faster progression of OSL compared with patients with lower BMI. Meanwhile, Tang et al. (19) presented that 22.8% (68/298 segments) of OLF spontaneously disappeared and the BMI of these patients reduced from 23.7 kg/m^2^ to 23.4 kg/m^2^ within the 5-year follow-up findings (*P* < 0.01); hence, OSL will likely instinctively improve and even disappear in nonobese patients. Notably, only one study assessed the severity of OSL based on the clinical symptoms instead of radiological data (8); however, previous studies have shown that evident symptoms are absent in a specific proportion of OSL cases (35). Therefore, future studies can focus on determining whether a high BMI can affect the severity of clinical symptoms in OSL.

The strengths of this systematic review and meta-analysis include the novelty of this topic, large population, strict methodology, and comprehensive subgroups. However, the current study also presents the following limitations. First, the majority of included studies were case–control types without a prospectively designed algorithm; hence, selection bias of individuals was unavoidable. Second, the characteristics of non-OSL individuals differed significantly across all studies. For instance, non-OSL individuals were patients with lumbar spondylosis instead of healthy patients in Chang et al. (20). As a result, the lenient selection of participants may affect the results of the current study. Third, high heterogeneity among the included studies and the use of a random-effects model for the meta-analysis may have affected the accuracy of the results. Fourth, the applicability of our conclusion to other races may be limited because all studies were performed on Asian populations. However, it should be noted that real-world data indicated that OSL frequently occurs in Asian populations, especially in the East Asian context (3). Fifth, the majority of the included studies explored the association of the BMI value instead of obesity or nonobesity with the onset or severity of OSL; as a result, an optimal cutoff value of the BMI remains unknown for the prediction of onset and severity of OSL. Finally, a selection bias may be induced because all the included studies were published in English.

## Conclusion

High BMI values might be significantly associated with the onset and severity of OSL. The results of this study can provide new evidence and insights into the prevention and treatment of OSL. Future studies can focus on further exploring the underlying mechanism of a high BMI value in the pathogenesis and severity of OSL, the effect of high BMI on the onset of OSL in other races and male individuals, the relationship of BMI with the severity of clinical symptoms of OSL, and the optional cutoff value of BMI to predict the onset and severity of OSL.

## Data Availability

The original contributions presented in the study are included in the article/**Suplementary Material**, further inquiries can be directed to the corresponding author/s.
