# Supplementary material for: High Body Mass Index Is Associated with an Increased Risk of the Onset and Severity of Ossification of Spinal Ligaments
Source: Front Surg. 2022 Jul 22;9:941672. doi: 10.3389/fsurg.2022.941672 (PMC9354543; doi:10.3389/fsurg.2022.941672)
Supplement: Supplementary file 1 [file Table_1_v1.docx]

**Supplementary Table 1 Details of literature search in PubMed database.**

| **Search number** | **Query** | **Results (n)** |
| --- | --- | --- |
| #1 | ossification of yellow ligament | 116 |
| #2 | ossification of ligamentum flavum | 564 |
| #3 | ossification of posterior longitudinal ligament | 2,144 |
| #4 | OPLL | 1,301 |
| #5 | ossification of the spinal ligament | 2,184 |
| #6 | #1 OR #2 OR #3 OR #4 OR #5 | 2,922 |
| #7 | body mass index | 284,231 |
| #8 | BMI | 176,917 |
| #9 | obesity | 421,764 |
| #10 | #7 OR #8 OR #9 | 632,850 |
| #11 | #6 AND #10 | 104 |
