# Supplementary material for: High Body Mass Index Is Associated with an Increased Risk of the Onset and Severity of Ossification of Spinal Ligaments
Source: Front Surg. 2022 Jul 22;9:941672. doi: 10.3389/fsurg.2022.941672 (PMC9354543; doi:10.3389/fsurg.2022.941672)
Supplement: Supplementary file 2 [file Table_2_v1.docx]

**Supplementary Table 2 Details of Newcastle-Ottawa Scale of included studies.**

| **Study** | **Was the case definition adequate** | **Representativeness of the cases** | **Selection of controls** | **Definition of controls** | **Comparability** | **Ascertainment of exposure** | **Same method of ascertainment for cases and controls** | **Non-response rate** | **Total score** |
| --- | --- | --- | --- | --- | --- | --- | --- | --- | --- |
| Shingyouchi 1996 (17) | 1 | 1 | 0 | 1 | 0 | 1 | 1 | 1 | 6 |
| Shirakura 2000 (18) | 1 | 1 | 0 | 0 | 1 | 1 | 1 | 1 | 6 |
| Kobashi 2004 (6) | 1 | 1 | 0 | 0 | 1 | 1 | 1 | 1 | 6 |
| Ikeda 2011 (11) | 1 | 1 | 0 | 1 | 0 | 1 | 1 | 1 | 6 |
| Mori 2014 (15) | 1 | 1 | 0 | 1 | 0 | 1 | 1 | 1 | 6 |
| Feng 2018 (12) | 1 | 1 | 0 | 1 | 1 | 1 | 1 | 1 | 7 |
| Kim 2018 (13) | 1 | 1 | 0 | 1 | 1 | 1 | 1 | 1 | 7 |
| Chang 2020 (20) | 1 | 1 | 0 | 1 | 1 | 1 | 1 | 1 | 7 |
| Liao 2020 (14) | 1 | 1 | 0 | 1 | 0 | 1 | 1 | 1 | 6 |
| Oshima 2020 (16) | 1 | 1 | 0 | 1 | 0 | 1 | 1 | 1 | 6 |
| Endo 2021 (9) | 1 | 1 | 0 | 1 | 0 | 1 | 1 | 1 | 6 |
| Tang 2021 (19) | 1 | 1 | 0 | 1 | 0 | 1 | 1 | 1 | 6 |
| Akune 2001 (7) | 1 | 1 | 0 | 1 | 0 | 1 | 1 | 1 | 6 |
| Hirai 2016 (10) | 1 | 1 | 0 | 1 | 1 | 1 | 1 | 1 | 7 |
| Ando 2017 (8) | 1 | 1 | 0 | 1 | 1 | 1 | 1 | 1 | 7 |
